# Broadly Neutralizing Antibodies for HIV-1 Prevention

**DOI:** 10.3389/fimmu.2021.712122

**Published:** 2021-07-20

**Authors:** Stephen R. Walsh, Michael S. Seaman

**Affiliations:** ^1^ Division of Infectious Diseases, Brigham and Women’s Hospital, Harvard Medical School, Boston, MA, United States; ^2^ Center for Virology and Vaccine Research, Beth Israel Deaconess Medical Center, Harvard Medical School, Boston, MA, United States

**Keywords:** HIV-1, antibody, neutralizing, Fc effector function, clinical trial

## Abstract

Given the absence of an effective vaccine for protection against HIV-1 infection, passive immunization strategies that utilize potent broadly neutralizing antibodies (bnAbs) to block acquisition of HIV-1 are being rigorously pursued in the clinical setting. bnAbs have demonstrated robust protection in preclinical animal models, and several leading bnAb candidates have shown favorable safety and pharmacokinetic profiles when tested individually or in combinations in early phase human clinical trials. Furthermore, passive administration of bnAbs in HIV-1 infected individuals has resulted in prolonged suppression of viral rebound following interruption of combination antiretroviral therapy, and robust antiviral activity when administered to viremic individuals. Recent results from the first efficacy trials testing repeated intravenous administrations of the anti-CD4 binding site bnAb VRC01 have demonstrated positive proof of concept that bnAb passive immunization can confer protection against HIV-1 infection in humans, but have also highlighted the considerable barriers that remain for such strategies to effectively contribute to control of the epidemic. In this review, we discuss the current status of clinical studies evaluating bnAbs for HIV-1 prevention, highlight lessons learned from the recent Antibody Mediated Prevention (AMP) efficacy trials, and provide an overview of strategies being employed to improve the breadth, potency, and durability of antiviral protection.

## Introduction

Human immunodeficiency virus (HIV) remains a major global health concern, with the World Health Organization (WHO) estimating that in 2019 there were 38 million HIV-1 infected individuals worldwide, 1.7 million new HIV infections, and 690,000 deaths from AIDS-related illness (1). Despite significant efforts over the past several decades, the development of an effective prophylactic vaccine against HIV-1 has yet to be realized. Thus, alternative biomedical strategies to prevent the transmission of HIV-1 are being actively pursued. Historically, passive immunization of pathogen-specific antibodies has proven to be an effective tool in the field of infectious disease for providing immediate yet transient protective immunity (2, 3). Recent advances in monoclonal antibody (mAb) engineering and production have accelerated the use of antibodies in the clinic for the treatment of cancers, autoimmune disease, and for targeting infectious pathogens or pathogen-derived toxins (4, 5). Early attempts to utilize passive infusion of HIV-1-specific neutralizing mAbs for treatment of infected individuals demonstrated little if any clinical benefit (6, 7). Given the limited breadth and potency of these first generation neutralizing antibodies, enthusiasm for a passive immunization strategy for HIV-1 prevention or treatment largely waned.

As new standardized and high-throughput assay platforms were developed for measuring neutralizing antibody activity against HIV-1 (8, 9), it became possible to screen and identify rare HIV-1 infected individuals whose serum neutralizing activity exhibited exceptional potency and breadth when tested *in vitro* against genetically diverse strains of virus (10–13). Using newly developed technologies to single-cell sort antigen specific B cells and clone antibodies from such individuals (14), the floodgates opened for the discovery of a second generation of highly potent broadly neutralizing antibodies (bnAbs) against HIV-1 (15–21). All bnAbs target HIV-1 Envelope (Env) which is expressed as a trimer of glycoprotein 120 (gp120) – gp41 heterodimers, and is the only target exposed on the surface of the virion. Through epitope mapping and refined structural studies of bnAb-Env complexes, it has been possible to identify major sites of vulnerability on HIV-1 Env that are primarily targeted by these new generation bnAbs. These include the CD4 binding site (CD4bs), the V3-glycan super site, the V2-glycan epitope on the apex of the trimer, the membrane-proximal external region (MPER) on gp41, and an epitope at the interface of the gp120 and gp41 subunits. Details regarding these antibodies and their epitope targets have been extensively reviewed elsewhere (22, 23). Here, we provide an overview of the preclinical development of bnAbs, the current status of bnAb clinical prevention trials, and areas for future development.

## bnAb-Mediated Protection in Animal Models

Both the humanized-mouse and non-human primate (NHP) animal models have been extensively used to characterize and model the protective-efficacy of bnAbs. The humanized mouse model relies on the reconstitution of immunodeficient mice with human hematopoietic and lymphoid cells, thus allowing for active HIV-1 replication and the ability to test investigational bnAbs for preventative or therapeutic potential with human immune cells (24). This model has been useful for initial proof-of-concept studies to demonstrate potent anti-viral protection *in vivo* by the newer generation bnAbs when delivered either as single antibodies, bnAb combination cocktails, or by vector-mediated expression using recombinant adeno-associated viruses (AAVs) (25–29). The potential utility of bnAbs for therapeutic treatment strategies has also been demonstrated using humanized mice. Treatment of animals with established HIV-1 YU2 infection using single bnAbs (PG16, 45-46^G54W^, PGT128, 3BC176 or 10-1074) resulted in a transient decline in viremia that ultimately rebounded, in part through the development of escape mutations that arose from bnAb-induced selective immune pressure (27). In contrast, treatment of infected animals with a combination mixture of the five bnAbs resulted in sustained suppression of viral replication for up to 60 days without evidence of escape, thus providing the first evidence that combinations of bnAbs may be required for effective control of virus for therapeutic treatment strategies. While the humanized mouse model has thus far been useful for pre-clinical proof-of-concept studies, there are several potential disadvantages that limit its utility for assessing bnAb efficacy. These include, in part, incomplete immune reconstitution, the lack of a robust innate and adaptive immune repertoire (the former being critical for assessing Fc-mediated effector functions of bnAbs), and the frequent development of xenogeneic graft *vs* host responses that limit the lifespan of the host (24, 30). Continued efforts to optimize the humanized mouse model, especially in regards to the evaluation of Fc-mediated effector functions, will help improve its utility.

NHP provide a more relevant model of human infection as they have an intact innate and adaptive immune repertoire and can be infected with chimeric simian-human immunodeficiency viruses (SHIVs) that express HIV-1 Envelope on an SIV backbone. Repeated low-dose mucosal challenge models have also been developed in NHP to better mimic natural HIV-1 transmission events. Passive immunization of bnAbs in NHP prior to challenge has been an effective model to elucidate the protective efficacy of bnAbs in the setting of intravenous, intrarectal, intravaginal, penile, and oral SHIV infection (31, 32). bnAbs currently in advanced clinical development targeting the CD4 binding site (VRC01, 3BNC117, VRC07-523.LS), the V3-glycan site (PGT121, 10-1074), the V2-glycan site (PGDM1400, CAP256-VRC26.25) and MPER epitope (10E8) have all demonstrated various levels of protection against infection in SHIV challenge models (33–38). The level of protection observed has strongly correlated with the potency of the bnAb against the specific strain of SHIV used for challenge and the durability of serum bnAb levels. A study comparing three bnAbs targeting either the CD4bs (VRC01 and 3BNC117) or V3-glycan epitope (10-1074) demonstrated that a single infusion dose of 20 mg/kg could afford protection from weekly low-dose intrarectal challenges with SHIV_AD8_ for up to 23 weeks (33). Animals receiving 3BNC117 infusion were protected for a median of 13 weeks, whereas those receiving VRC01, which is less potent against the SHIV_AD8_ challenge virus, were only protected for a median of 8 weeks. A comparison of high-dose mucosal SHIV challenge studies using six bnAbs targeting the CD4bs or V3-glycan epitopes used regression analysis of the combined dataset to estimate that a serum 50% inhibitory dilution (ID_50_) titer of 1:100 was sufficient to prevent acquisition in 50% of NHP (35). These findings were further supported by a more recent meta-analysis of data from 13 bnAb protection studies utilizing 274 NHP passively infused with 16 different bnAbs and eight strains of SHIV which demonstrated that serum neutralization titer on the day of SHIV challenge was strongly associated with protective efficacy against the challenge virus (32). Logistic modeling that adjusted for bnAb epitopes and challenge viruses estimated that serum ID_50_ titers of 55, 219, and 685 would be required to achieve 50%, 75%, or 95% protection, respectively. These analyses support the hypothesis that serum neutralization titer against the infecting strain of HIV-1 will be a key determinant of protection for bnAb prevention strategies in humans, and further highlight the possible requirement for substantial serum neutralization titers at the time of exposure for effective sterilizing immunity.

While NHP have been instrumental for investigating the potential protective efficacy of bnAbs for both HIV-1 prevention and therapeutic treatment strategies, there are several limitations to this model that should be taken into consideration. These include in part: (i) the limited genetic diversity of SHIVs compared to circulating strains of HIV-1 that are encountered in nature, (ii) the higher per exposure infection rate in SHIV challenge models compared to natural HIV-1 infection in humans, (iii) potential inefficiencies of human bnAbs to engage and activate innate immune effector functions in NHP, and (iv) the interference of anti-drug antibody (ADA) responses that often arise when NHPs elicit an autologous antibody response against the passively infused human bnAb (33, 39–43). Despite these potential drawbacks, NHP remain the best available animal model for bnAb protection studies. Recent advances in the development of genetically diverse panels of SHIVs that recapitulate many features of HIV-1 infection will certainly allow for more rigorous assessment of protection afforded by bnAbs and bnAb combinations (44–47). Together, both the humanized mouse and NHP models have been critical for accelerating the translation of bnAb passive immunity into human clinical trials.

## Effector Mechanisms

Animal model studies have demonstrated that both Fab-mediated neutralization of virus and Fc-dependent effector functions can contribute to antiviral protection of bnAbs. The Fc-mediated component may be especially important for elimination of virus-infected cells and preventing the establishment of chronic infection (48–50). This may occur *via* binding to Fcγ-receptors (FcγRs) expressed on NK cells, macrophages and neutrophils to activate innate effector mechanisms such as antibody-dependent cellular cytotoxicity (ADCC) and antibody-dependent cellular phagocytosis (ADCP), or by activation of antibody-dependent complement-mediated lysis. Indeed, it has been demonstrated that passive protection afforded by PGT121 against a mucosal SHIV challenge in NHP involved clearance of disseminated virus in distal tissues rather than a complete blockade of virus at the mucosal surface (51). Elimination of these productively infected distal foci was hypothesized to involve Fc-effector mechanisms given evidence of innate immune activation in these tissues. Passive administration of bnAbs 3BNC117 and 10-1074 have also been shown to accelerate the clearance of HIV-1 infected CD4+ T cells in humanized mice through an FcγR-dependent mechanism (52).

Direct evaluation of the relative protective contribution of Fab-mediated neutralization versus Fc-mediated effector functions *in vivo* has also been investigated using antibodies with site-directed mutations to knock-out binding to FcγRs and/or complement. Results from such studies, however, have often come to conflicting conclusions. Hessell et al. first demonstrated the potential importance of Fc-mediated effector functions in NHP when protection afforded by passive immunization of b12, a first generation neutralizing mAb, was partially abrogated by elimination of Fc-receptor binding through the L234A/L235A (LALA) mutation (53). No reduction in protective effect was observed with a b12 variant that lacked complement binding (K332A). In contrast, two recent studies in NHP investigating the contribution of Fc effector functions with bnAb PGT121 in the setting of passive protection against SHIV_SF162P3_ challenge (both cell-free and cell-associated viral infection models) found no evidence that effector functions contributed to bnAb-mediated protection, even when PGT121 wildtype and Fc-knock out variants were administered at sub-protective doses (54, 55). Furthermore, partial depletion of NK cells which are key mediators of Fc-dependent effector functions did not abrogate the protective efficacy of PGT121 (55).

Two recent studies utilized knowledge of HIV-1 dynamics and mathematical modeling to quantify the contribution of Fc-mediated effector functions to the antiviral activity of bnAbs in the context of therapeutic treatment of an established infection. By measuring the kinetics of viral load decline in response to a single dose treatment with either a wildtype bi-specific bnAb (composed of Fabs from 3BNC117 and PGDM1400) or a matched Fc-null variant, Wang and colleagues observed an earlier and sharper decline in viral load in both HIV-1-infected humanized mice and SHIV-infected NHP when treated with wildtype bnAb compared to the variant with deficient Fc effector function (56). Quantification of these observed differences suggested that Fc-mediated effector functions accounted for 25-45% of antiviral activity. Using similar methods, Asokan et al. observed that Fc-mediated effector functions contributed 21% of the anti-viral activity of bnAb VRC07-523-LS when infused into viremic SHIV-infected macaques (57). Interestingly, several studies that have examined passive administration of bnAb variants designed for enhanced binding to FcγRs, and therefore hypothesized to elicit greater ADCC activity, in fact observed no augmentation of protection in SHIV-infected NHP (57, 58).

Together, these data suggest that the predominant mechanism of bnAb-mediated protection is through direct neutralization of virions, but that Fc-mediated effector functions can contribute to overall protective efficacy. The latter may be more critical with the use of bnAbs in therapeutic settings in which virally infected cells must be targeted for elimination. Understanding whether individual bnAbs or bnAb-epitope classes differentially exhibit abilities to recruit Fc-mediated effector functions *in vivo* and whether these activities can be enhanced through Fc variant engineering will be important for exploring improved antibody-based strategies for HIV-1 prevention or therapeutic treatment (59).

## Combinations of bnAbs

HIV-1 Env sequence diversity remains a major challenge for bnAb passive prevention strategies, as individuals may be exposed to highly diverse swarms of virus that exhibit a range of neutralization sensitivities or complete resistance to any particular antibody. Furthermore, clade-specific resistance patterns have been identified for certain bnAbs which may complicate the use of these antibodies in geographical regions where these particular HIV-1 subtypes dominate. For example, CRF01_AE is the major circulating lineage in Southeast Asia, and viruses in this clade exhibit high level resistance to bnAbs targeting the V3-glycan epitope, such as PGT121 and 10-1074, due to the loss of the critical N332 glycosylation site (60). Similarly, clade B viruses demonstrate higher levels of resistance to bnAbs targeting the V2-glycan epitope, such as PGDM1400 and CAP256, compared to clade C viruses (60, 61). It is therefore likely that the clinical success of bnAb passive immunization strategies will require combinations of antibodies to increase the overall breadth and potency against diverse isolates and to prevent the emergence of resistance. A central question then arises as to which bnAbs and how many will be required to provide optimal protection. Mathematical modeling approaches have been developed that utilize *in vitro* neutralization data of clinically advanced bnAbs tested against large panels of genetically diverse HIV-1 Env pseudoviruses to help determine optimal combinations (62, 63). These analyses have demonstrated that combinations containing three or four bnAbs targeting different epitopes typically act additively to provide better breadth, potency, and extent of complete neutralization compared to two antibody combinations, and further increase the probability of having multiple bnAbs simultaneously active against a given virus. Combinations of bnAbs targeting independent epitopes are also more favorable than those containing antibodies targeting overlapping epitopes (e.g., two or more bnAbs targeting the CD4bs). Given that subtype-specific resistance patterns of bnAbs should be considered in decisions to test bnAbs in particular geographical regions, defining optimal combinations for clade-specific or regional use is an area of active investigation (64).

The optimal combination and number of bnAbs needed may also vary depending on the intended clinical use. For prevention of HIV-1 acquisition, it is possible that optimal combinations of two or three bnAbs may achieve sufficient breadth and potency to ensure reliable coverage against the transmitting virus. For therapeutic treatment strategies however, it is likely that larger numbers of bnAbs will be required to adequately cover the within-host diversity that exists as replicating virus or is present in the latent reservoir. The ability to accurately screen HIV-infected individuals to determine bnAb sensitivity profiles and compose optimal combinations to target the patient-specific viral quasispecies would be beneficial, similar to genotyping strategies used for optimizing combination antiretroviral drug regimens. While phenotyping the bnAb sensitivity of patient viruses can be performed using either bulk or limiting dilution T cell outgrowth cultures, these assays can be labor intensive, costly, and may fail to detect minor pre-existing resistant variants (65, 66). An alternative strategy for future development may be to use predictive modeling based on *env* sequencing of the patient’s quasispecies to determine bnAb sensitivity patterns and optimize combination cocktails (67–69).

Combination bnAb regimens will also have additional complexities in manufacturing, product development, and clinical implementation that will need to be addressed. For example, each bnAb will have its own unique pharmacokinetic profile which may present challenges in formulation and delivery in order to maintain all antibodies in the combination above target therapeutic concentrations *in vivo* for optimal protective efficacy. Further development of multiple antibodies in a single co-formulated drug product will also need to take into account the specific formulation and stability characteristics of each bnAb. Recent reports have demonstrated that protein sequence optimization of several clinically advanced bnAbs can improve expression levels, conformational stability, and downstream processing and formulation conditions, all while maintaining the neutralization profile of the parental antibody (70, 71). A high concentration co-formulated drug product containing bnAbs 3BNC117-LS and 10-1074-LS that will allow for subcutaneous administration has also recently been described (72). As will be reviewed further below, the first 2- and 3-bnAb combinations have initiated clinical testing in HIV-infected and uninfected individuals. With the continued development of methods for predicting optimal bnAb combinations and improvement of manufacturing capabilities it is expected that the portfolio of bnAb combinations entering human clinical trials will continue to expand.

## Clinical Evaluation of bnAbs

The availability of clinical grade bnAbs capable of blocking HIV-1 *in vitro* and in animal challenge models has opened the possibility of antibody-mediated prevention (AMP) of HIV infection in humans (41). Several bnAbs have been tested in phase 1 studies to determine their safety, tolerability, and pharmacokinetic (PK) profiles in both HIV-infected and HIV-uninfected individuals (**Table 1**). Thus far, only the CD4bs targeting bnAb VRC01 has advanced to efficacy studies for protection against HIV infection, but ongoing combination bnAb trials will likely inform future efforts.

**Table 1:** bnAbs in Clinical Trials.

Epitope region targeted	bnAb	Half-life	Reference
CD4-binding site (CD4-BS)	VRC01	15 days; 71 days (-LS)	NCT02165267 (73, 74);
	VRC07-523	40 days (-LS)	NCT03015181 (75, 76);
	3BNC117	18 days; 61 days (-LS)	NCT02018510 (77, 78);
	N6	>30 days (-LS)	NCT03538626 (79);
Membrane-proximal external region (MPER)	10E8	N/A	NCT03565315 (80);
V1V2 loop glycan	PGDM1400	N/A	NCT03205917
	PG9^1^	N/A	NCT01937455 (81)
	CAP256-VRC26.25	N/A	PACTR202003767867253 (82);
V3 loop glycan	PGT121	22 days	NCT02960581 (83);
	10-1074	24 days; 73.5 days (-LS)	NCT02511990 (78, 84);
gp120 outer domain	2G12	16.5 days^2^	(85)

^1^AAV delivery system.

^2^tested in HIV-infected participants.

NA, Not available.

### CD4 Binding Site bnAbs

#### VRC01

VRC01 was originally discovered in an individual infected with HIV-1 for more than 15 years and whose immune system controlled the virus without antiretroviral therapy (ART) (20, 86). When tested *in vitro* against a genetically diverse panel of 190 HIV-1 Env pseudoviruses, VRC01 neutralized 91% with a 50% inhibitory concentration (IC_50_) of < 50 μg/ml and 72% with an IC_50_ < 1 μg/ml.

Clinical studies of VRC01 started in 2013, first in HIV-infected adults [VRC 601 (NCT01950325) (87)], and then in healthy adults [VRC 602 (NCT01993706) (88] and HVTN 104 (NCT02165267) (89)]. VRC01 was found to be safe and well tolerated at doses ranging from 5-40 mg/kg administered intravenously (IV) and at 5 mg/kg subcutaneously (SC). PK analyses across studies have shown that VRC01 has a half-life of approximately 15 days with little difference between dose groups. Importantly, VRC01 retained its expected neutralizing activity in participants’ serum and no ADA responses were detected over the course of multiple infusions. In addition, a study of VRC01 in infants born to HIV-1 infected mothers is ongoing [IMPAACT P1112 (NCT02256631)].

VRC01 was further tested in two similar clinical trials, ACTG A5340 [NCT02463227] and NIH 15-I-0140 [NCT02471326], which were reported together and in which fully suppressed patients on stable ART underwent an analytic treatment interruption (ATI) after receiving VRC01 at a dose of 40 mg/kg (90). Disappointingly, viral rebound occurred despite VRC01 serum concentrations above 50 μg/ml with a mean time to rebound of 4 to 6 weeks. VRC01 was also found to exert selection pressure on emergent viruses which raises concerns about the development of resistance during treatment or prophylactic use.

#### 3BNC117

3BNC117 is a more potent and broad CD4bs bnAb than VRC01 and was isolated from a chronically HIV-infected donor with high serum neutralization activity (21). In a first in human open-label study [NCT02018510], 3BNC117 was found to be safe and well-tolerated in HIV-infected participants and it was observed that a single IV dose of 30 mg/kg reduced viral loads of viremic participants for up to 28 days post-infusion (77). In HIV-uninfected participants, 3BNC117 is also safe and well tolerated across a range of doses *via* both the IV and SC routes and has a serum half-life of approximately 17 days (77, 91).

#### VRC07-523LS

VRC07 is a CD4bs targeting bnAb that was cloned from the same HIV-infected donor from whom VRC01 was isolated. The VRC07 heavy chain was identified by deep sequencing based on its similarity to VRC01 and paired with the VRC01 light chain (92). VRC07 was then engineered with a series of mutations (called 523) which increased the breadth and potency compared to the parental antibody. As had also been done to VRC01 (73), modifications to the antibody Fc domain were introduced to improve antibody half-life. A common two amino acid substitution (M428L/N434S, referred to as “LS”) increases the antibody’s binding affinity for the neonatal Fc-receptor (FcRn), resulting in increased recirculation of functional IgG (93, 94) thereby prolonging the *in vivo* half-life. *In vitro*, VRC07-523LS is 5-to 8-fold more potent than VRC01, as well as broader, with an IC_50_ < 1 μg/ml against 92% of HIV-1 pseudoviruses from diverse clades (92).

A phase 1, first in human dose-escalation study of VRC07-523LS, [VRC 605 (NCT03015181)] has been completed in healthy, HIV-uninfected adults and evaluated the safety, tolerability, and PK of one to three administrations of the antibody (75). The doses evaluated ranged from a single administration of 1 mg/kg to 40 mg/kg IV, as well as three administrations (every 90 days) of 5 mg/kg SC and 20 mg/kg IV. VRC07-523LS was found to be safe and well-tolerated with a promising PK profile.

The HVTN 127/HPTN 087 trial (NCT03387150), which opened in February 2018, enrolled a total of 144 healthy, HIV-uninfected adult participants who received multiple injections of VRC07-523LS administered *via* IV, SC, and IM routes at a range of doses. The primary objectives are to assess safety and tolerability of repeated IV, SC, or IM administrations of VRC07-523LS as well as to characterize serum levels over time for the different doses and routes of administration. Additional objectives include building a population PK model of VRC07-523LS, and determining if ADA emerge in response to repeated administrations. A companion study, HVTN 128 (NCT03735849), is assessing the PK of VRC07-523LS in mucosal secretions (semen, cervical secretions, and rectal mucous) and mucosal biopsies (rectal, cervical, and vaginal tissue biopsies). Both studies have completed all follow-up visits and are in the analysis phase. Preliminary data from HVTN 127/HPTN 087 were presented at the virtual R4P conference in January 2021 and it was reported that VRC07-523LS was safe and well-tolerated with a serum half-life of about 40 days (76).

#### N6

Another, even broader CD4bs targeting bnAb has been isolated from an HIV-infected donor found to have potent neutralizing serum activity and is called N6 (95). *In vitro*, N6 was found to neutralize 98% of 181 pseudoviruses at an IC_50_ < 50 μg/ml, including several isolates which are resistant to other VRC01-class bnAbs. Overall, N6 had a median IC_50_ of 0.038 μg/ml. N6 was synthesized as an IgG1 with the –LS mutation to increase its *in vivo* half-life and administered in a first in human study [VRC 609 (NCT03538626)] at doses ranging from 5 mg/kg to 40 mg/kg. Preliminary data were presented at CROI 2020 and it was reported that N6LS was safe and well-tolerated with a half-life exceeding 30 days (79).

### V3 Glycan bnAbs

#### PGT121

PGT121 was originally isolated from an HIV-infected donor in the IAVI Protocol G African cohort and was found to target the V3 glycan region on the gp120 Env protein. This epitope includes both peptide and glycan elements and is centered on the conserved residue N332 (96, 97). Although its coverage is somewhat less broad when assessed in *in vitro* pseudovirus neutralization assays (63% for PGT121 *vs.* 93% for VRC01 at an IC_50_ < 50 μg/ml), PGT121 has about 10-fold higher median neutralizing potency than VRC01 and about 100-fold higher potency than first generation mAbs such as 2G12, b12, or 4E10 (18).

PGT121 has been tested in a phase 1 study called T001 which was a randomized, placebo-controlled trial of the safety, PK, and antiviral activity of this bnAb in both HIV-uninfected and HIV-infected adults [NCT 02960581]. IV infusions and SC injections were found to be safe and well-tolerated in all participants (83). The median half-life of PGT121 during the elimination phase was approximately 23 days (range 19 to 26 days).

#### 10-1074

10-1074 was isolated from the same clade A infected African donor as PGT121 (13). Like PGT121, 10-1074 recognizes an epitope on the gp120 V3 outer domain that includes both peptides and glycans and is centered on the conserved amino acid residue N332 (98, 99). When tested against large panels of HIV-1 pseudoviruses *in vitro*, 10-1074 neutralized 65% of 306 strains comprising 13 subtypes with an average 80% inhibitory concentration (IC_80_) titer of 0.18 μg/ml (84).

10-1074 has been tested in both HIV-infected and HIV-uninfected participants and found to be safe and well-tolerated at doses ranging from 3 mg/kg to 30 mg/kg [NCT02511990] (84). Amongst HIV-infected individuals with detectable viremia, 10-1074 infusion was found to induce a rapid decrease in plasma viral load, with one participant having a virologic response that was sustained for 58 days following a 30 mg/kg IV infusion.

### V2 Glycan bnAbs

#### PGDM1400

PGDM1400 was isolated from another HIV-infected donor from the IAVI Protocol G African cohort and was found to interact with glycans in the region of residue N160 on the V2 loop of gp120 Env (100). PGDM1400 binding is highly quaternary-structure dependent and this bnAb is exceptionally potent, with 83% coverage of a globally representative panel of pseudoviruses at a median IC_50_ of 0.003 μg/ml. Compared to three of the CD4bs antibodies (VRC01, VRC07, and 3BNC117), PGDM1400 is 10 to 100-fold more potent (18, 21, 100).

PGDM1400 has been tested in a phase 1 study called T002 which is a phase 1 randomized placebo-controlled clinical trial of the safety, pharmacokinetics, and antiviral activity of this bnAb in both HIV-uninfected and HIV-infected adults. [NCT03205917]. This study also tested the dual combination of PGDM1400 + PGT121 and a triple combination with the addition of VRC07-523LS. Results from this study are expected soon.

#### CAP256

Another V2 loop targeting bnAb was isolated from an individual in the Centre for the AIDS Programme of Research in South Africa (CAPRISA) 002 Acute Infection study (101). This patient was infected with a clade C isolate and then superinfected with another clade C isolate and found to have broad serum neutralization activity (102, 103). One bnAb isolated was called CAP256-VRC26.25 and found to be exceptionally potent, especially against clade C isolates which predominate in southern Africa (62). A variant of this bnAb (CAP256V2LS) has been modified slightly to improve manufacturability and to include the –LS mutation, and is being tested in humans in a single-center phase 1 clinical trial called CAPRISA 012 [PACTR202003767867253] (82). Subsequent groups in CAPRISA 012 will evaluate CAP256V2LS in combination with VRC07-523LS and/or PGT121 (82).

### MPER bnAbs

#### 10E8

Amongst the broadest in terms of overall global viral coverage are MPER targeting bnAbs. 10E8, like other bnAbs, was isolated from an HIV-infected donor with high serum neutralization activity and was found to bind to the same MPER gp41 epitopes as 4E10 (104). *In vitro* studies found that 10E8 did not bind phospholipids and did not bind to HEp-2 cells or a panel of autoantigens (104), unlike other MPER-targeting antibodies. A series of variants of 10E8 were developed for increased solubility as well as manufacturing ease (105). In NIH 18-I-0113 [NCT03565315], a leading candidate that had been engineered for a longer half-life (with the –LS mutation), 10E8VLS, was found to have a disappointing PK profile in terms of *in vivo* half-life. More concerning, however, was that one of the eight participants who received a single SC injection of 5 mg/kg 10E8VLS experienced severe injection site erythema and was found to have panniculitis. This led to the suspension of the study (80) and may have been due to a lipid-binding cross-reaction, although the mechanism remains under investigation.

### AMP Efficacy Trials

As VRC01 was found to be safe and well-tolerated in healthy HIV-uninfected volunteers and had modest anti-viral effects in viremic HIV-infected participants, an important question was whether VRC01 could prevent HIV infection (106). The NIAID-funded HIV Vaccine Trials Network (HVTN) and HIV Prevention Trials Network (HPTN) therefore collaborated on the design and conduct of the two Antibody Mediated Prevention (AMP) trials, HVTN 704/HPTN 085 (NCT02716675) and HVTN 703/HPTN 081 (NCT02568215) (107). HVTN 704/HPTN 085 enrolled 2,699 cisgender men and transgender (TG) people who have sex with men in Brazil, Peru, Switzerland, and the US (108) while HVTN 703/HPTN 081 enrolled 1,924 cisgender women in Botswana, Kenya, Malawi, Mozambique, South Africa, Tanzania, and Zimbabwe (109).

The underlying hypothesis of the coordinated AMP trials was that passive transfer of an HIV-1 bnAb would be efficacious at preventing sexual transmission of HIV-1 in exposed individuals. A key secondary endpoint was to determine if an association existed between serum neutralization activity and the ID_50_ or ID_80_ titers required for protection as had been shown in NHP/SHIV models (41, 110). Based on the NHP models, it was estimated that protection would be achieved at serum antibody concentrations 50-100-fold higher than the measured IC_50_ of the challenge (infecting) virus (32). Based on *in vitro* neutralization profiles of panels of HIV-1 isolates, it was anticipated that between 65% (clade C) and 81% (clade B) of infecting viruses would be susceptible to VRC01.

The two trials were each designed with 90% power to detect prevention efficacy (PE) of 60% comparing the two VRC01 groups *vs* the placebo group, based on an assumption of an annual background HIV-1 incidence of 5.5% in HVTN 703/HPTN 081 and 3% in HVTN 704/HPTN 085 and a dropout rate of 10% of participants per year (107). Participants were randomly assigned to one of three groups at a 1:1:1 ratio of VRC01 10 mg/kg, VRC01 30 mg/kg, or placebo. IV administration of VRC01 or placebo occurred every eight weeks for a total of 10 infusions. Pre-exposure prophylaxis (PrEP) was encouraged by the trial investigators and access to emtricitabine (FTC)/tenofovir disoproxil fumarate (TDF) was facilitated by the study.

Study conduct was very high quality. Loss to follow-up was low (9.4% per year in HVTN 704/HPTN 085 versus 6.3% per year in HVTN 703/HPTN 081) and 79% of participants in HVTN 704/HPTN 085 and 76% in HVTN 703/HPTN 081 received all 10 infusions (74). Rates of PrEP usage differed considerably between the two studies demonstrating some of the challenges in increasing PrEP uptake. Effective concentrations of FTC/TDF were detected in 28.9% of person-years in HVTN 704/HPTN 085 but only 0.4% of person-years in HVTN 703/HPTN 081 (74).

As had been noted in the Phase 1 studies, VRC01 infusions were safe and generally very well-tolerated. Moderate to severe adverse events which were deemed related to VRC01 were noted in 1.2% of participants in HVTN 704/HPTN 085 versus 3.0% of the participants in HVTN 703/HPTN 081. Infusion reactions were seen in a small number of participants and were generally mild to moderate, and typically resolved quickly (111).

The overall results from the AMP trials, however, were a disappointment. At week 80, the PE for the combined VRC01 groups *versus* placebo was 26.6% in HVTN 704/HPTN 085 and 8.8% in HVTN 703/HPTN 081 (p=0.15 *vs* placebo for HVTN 704/HPTN 085 and p=0.70 for HVTN 703/HPTN 081, respectively) (74). PE did not differ significantly by dose in either study. In HVTN 704/HPTN 085, PE was 22.4% in the low dose VRC01 group versus 30.9% in the high dose group, compared with –9.3% in the 10 mg/kg group, and 27.0% in the 30 mg/kg group in HVTN 703/HPTN 081 (74). While the point estimates of PE for the higher dose VRC01 groups are similar to the vaccine efficacy reported in the RV144 trial [31.2% (112)], the 95% confidence intervals (CIs) of the PEs in the AMP studies were quite large and all crossed the null.

Secondary analyses, however, demonstrated that for infecting HIV-1 isolates that were highly sensitive to VRC01 neutralization (prospectively defined as an IC_80_ <1 μg/mL) protective efficacy was high, with an estimated PE of 75.4% and 95% CI from 45.5% to 88.9% (74). The majority of infecting HIV-1 isolates had IC_80_ > 1 μg/mL (55% had IC_80_ > 3 μg/mL) and there was no statistically significant protective efficacy seen against these more resistant viruses (74). Taken together, the infecting viruses in the combined VRC01 groups had a geometric mean IC_80_ of 8.4 μg/mL versus 3.5 μg/mL in the placebo group (74), suggesting there was selection pressure on infecting viruses by VRC01 during acquisition. This immunologic selection pressure was also found to have an effect on viremia at the time of diagnosis amongst VRC01 recipients who were infected with highly sensitive strains HIV-1 with geometric mean viral loads of 9,800 copies/mL compared to 176,000 copies/mL for placebo recipients (74). This effect of mAb administration was not seen amongst participants infected with more resistant HIV-1 isolates.

These data provide several important lessons to help guide future trials. The AMP studies successfully enrolled at-risk participants and despite the complexities of intravenous infusions every two months, retention and engagement was high throughout the study. They also demonstrated the key proof-of-principle that a bnAb can prevent HIV infection in people. However, while VRC01 can prevent sexual HIV-1 acquisition, most circulating viruses have neutralization levels high enough to make them essentially resistant. Furthermore, *in vitro* neutralization sensitivity is a useful biomarker for preventive efficacy *in vivo*, although the threshold for protection (< 1 μg/mL) is considerably higher than had been predicted (10 μg/mL). These predictions were primarily based on *in vitro* testing of VRC01 against panels of HIV-1 Env pseudoviruses. Several studies have demonstrated that pseudoviruses produced *via* transfection of 293T cells are considerably more sensitive to neutralization by patient serum and bnAbs when compared to matched isolates expanded in peripheral blood mononuclear cells (PBMC) (113, 114). This point should be taken into consideration when incorporating *in vitro* neutralization data into clinical efficacy estimations for HIV-1 bnAbs. While bnAbs that are more potent and have broader coverage than VRC01 have been identified, it remains to be determined whether the threshold of protection identified by the AMP study will translate to other bnAbs targeting the CD4bs or other epitopes, or whether this threshold can be reliably achieved and maintained through repeated passive infusions. The AMP study suggests that a single monovalent bnAb is highly unlikely to have the breadth and potency required, but nonetheless, the results do provide a benchmark for future bnAb studies to build upon.

### Clinical Testing of bnAb Combinations

To address the significant challenges of breadth and potency, combinations of bnAbs are being tested in early phase studies (**Table 2**). As bnAbs which target different regions of the Env protein have been found to have additive effects on inhibition *in vitro* (64), the combinations moving forward are complementary with respect to coverage. This concept is supported by NHP challenge studies showing that a combination of 2 bnAbs fully protected macaques against a mixed SHIV challenge when neither mAb administered alone was able to do so (39).

**Table 2:** Selected Clinical Trials of bnAb Combinations.

Name	bnAbs	Env region targeted	Reference
IAVI T002^1^	PGT121	V3 glycan	NCT03205917 (83);
	PGDM1400	V1V2 glycan	
YCO-0899^2^	3BNC117	CD4-binding site	NCT02824536 (91);
MCA-0906^3^	10-1074	V3 glycan	NCT02825797 (115);
HVTN130/HPTN089	VRC07-523LS	CD4-binding site	NCT03928821
	PGT121 or 10-1074	V3 glycan	
	PGDM1400	V1V2 glycan	
HVTN136/HPTN092	VRC07-523LS	CD4-binding site	NCT04212091
	PGT121.141.LS	V3 glycan	
IAVI C100	3BNC117-LS-J	CD4-binding site	NCT04173819 (78);
	10-1074-LS-J	V3 glycan	
CAPRISA 012B	CAP256V2LS	V1V2 glycan	PACTR202003767867253 (82);
	VRC07-523LS	CD4-binding site	
	PGT121	V3 glycan	

^1^Included both HIV-infected and HIV-uninfected participants.

^2^HIV-uninfected participants.

^3^HIV-infected participants.

#### HVTN130/HPTN089

In HVTN130/HPTN089 [NCT03928821], combinations of two or three complementary bnAbs are being tested for safety and PK parameters. The engineered CD4-binding site mAb VRC07-523LS is the broadest of the mAbs in this study and has the longest half-life due to the –LS mutation. Individually, PGT121 and PGDM1400 display more limited breadth than VRC07-523LS, but together they exhibit complementary coverage against diverse HIV-1 strains. Furthermore, they are remarkably potent, having amongst the lowest median IC_80_ titers among the bnAbs identified to date (18). In silico modeling suggests that the triple combination of VRC07-523LS + PGT121 + PGDM1400 has >90% coverage of global isolates using a cutoff of ID_80_ of 1 μg/ml and >80% coverage with a cutoff of 0.1 μg/ml (62–64). 10-1074 is being tested in this protocol as an alternative V3 glycan bnAb to compare versus PGT121 in combination with VRC07-523LS. This study is fully enrolled and data are expected to be presented soon.

#### HVTN136/HPTN092

In HVTN136/HPTN092 [NCT04212091], an –LS variant of the V3 glycan targeting mAb PGT121 (PGT121.414.LS) is being tested alone and in a combination regimen, again with VRC07-523LS as the CD4bs targeting bnAb. The study is currently enrolling and the first groups will provide safety and PK data for PGT121.414.LS alone, which is being administered to people for the first time in this study, at doses ranging from 3 mg/kg to 30 mg/kg IV and 5mg/kg SC. The subsequent groups will combine the two mAbs at 20 mg/kg IV versus 5 mg/kg SC.

#### IAVI C-100

A small single-center phase 1 study which combined the 3BNC117 and 10-1074 bnAbs was conducted and demonstrated that doses ranging from 3 mg/kg to 10 mg/kg were safe and well-tolerated (91). Importantly, there appeared to be no difference in the half-lives of the two bnAbs compared to when they were administered alone. This bnAb combination was also shown to maintain suppression of viremia and to prevent the emergence of resistant variants in HIV-1 infected individuals undergoing ART interruption (116), and to significantly reduce viral loads in viremic patients harboring dual sensitive viruses for up to 3 months following the last infusion (115). To improve the PK profile of this combination, -LS variants of the two bnAbs have been engineered and are being tested in a study called IAVI C100 [NCT04173819]. This study will evaluate the safety, tolerability, and PK of 3BNC117-LS-J and 10-1074-LS-J administered alone and in combination *via* IV and SC routes (78). The study is currently enrolling.

## Future Directions

The results from the AMP trials highlight that bnAbs with exceptional levels of breadth, potency and *in vivo* durability will be needed to have an appreciable impact on the prevention of HIV-1 acquisition. Efforts continue to identify new bnAbs with enhanced neutralization profiles that may be viable for clinical development either as single drug products, or to complement existing bnAbs for use in combination cocktails (117–119). Antibody engineering strategies are also being employed to improve the breadth and potency of existing bnAbs. Structure-guided rational design modifications of the antigen binding Fab domains have been successfully used to enhance interactions with the Env trimer resulting in improved neutralizing activity (92, 120, 121). Increasing the *in vivo* half-life of antibodies can also be achieved through engineering modifications to the antibody Fc domain, most notably the –LS mutations discussed above (93, 94). Multiple bnAbs currently in clinical development are being tested with the LS mutations, and it is hoped that such improvements in the pharmacokinetic profiles of bnAbs may make it possible to achieve and maintain therapeutic levels with lower-dose and less frequent administrations. Strategies for persistent expression of bnAbs *in vivo* by vectored antibody gene delivery using rAAV have shown promise in humanized mouse and NHP models of infection (122), and one clinical trial has evaluated rAAV1 delivery of the V2-glycan targeting bnAb PG9 in humans (NCT01937455) (81). While this study demonstrated that rAAV delivery was safe and well tolerated, there was no detection of PG9 in serum by ELISA, and the development of ADA and anti-vector antibody responses was observed in several volunteers, thus highlighting the challenges that remain for developing such approaches. A second phase I trial assessing rAAV8 delivery of the CD4bs targeting bnAb VRC07 in HIV-1-infected adults on antiretroviral therapy is currently ongoing (NCT03374202). Several initial proof-of-concept studies in mice have also evaluated the approach of gene-editing primary B cells to express bnAb antibody receptors on the cell surface (123, 124). Gene-edited B cells passively infused into wildtype recipients have the capacity to respond to vaccination with cognate antigen resulting in clonal expansion, affinity maturation, expression of high levels of serum bnAb, and establishment of durable memory. Future studies using the NHP/SHIV model will be important for further evaluating the potential of such approaches to provide durable sterilizing protection.

HIV-1 Env sequence diversity remains a major obstacle for the clinical development of antibody-based prevention strategies. As previously discussed, clinical trials are ongoing to assess antibody cocktails containing two or three bnAbs targeting independent epitopes on the Env trimer to provide greater coverage against circulating isolates and to circumvent viral escape. A parallel strategy being actively explored is to engineer bi- or tri-specific antibodies that combine Fabs from different bnAbs onto the same antibody molecule (125). Multiple bi-specific antibodies targeting various epitope combinations have demonstrated enhanced breadth and potency compared to single bnAb molecules in *in vitro* testing (95, 126). For example, the bi-specific antibody 10E8.4/iMab which targets the MPER epitope of Env and the host CD4 molecule has demonstrated exquisite breadth and potency when tested against large multiclade panels of viruses (100% breadth with mean IC_50_ values of 0.002 μg/ml) and protection of humanized mice against HIV-1 infection (95). This molecule is currently being evaluated in a human clinical trial in HIV-1-infected and uninfected volunteers (NCT03875209). Another example is a tri-specific antibody that contains Fabs derived from the CD4bs antibody VRC01, the V2-glycan antibody PGDM1400 and the MPER antibody 10E8 (127). This antibody has also exhibited excellent neutralization breadth and potency *in vitro*, demonstrated complete protection in NHP against a mixed SHIV challenge, and is also currently being evaluated in a phase 1 human clinical trial (NCT03705169). Another strategy has been to engineer entry inhibitor molecules that target both the primary CD4 receptor and co-receptor binding sites on Env which are highly conserved across strains of HIV-1. One example in this class of molecules is eCD4-Ig which combines domains 1 and 2 of human CD4 fused to an IgG Fc with an attached sulfated CCR5-mimetic peptide. This molecule has also demonstrated 100% neutralization against all HIV-1, HIV-2, and SIV strains tested to date, and confers robust protection against SHIV and SIV challenges when delivered using rAAV vectors (128). While these strategies have theoretical benefits over cocktail combinations of individual bnAbs in regards to manufacturing and the complexity of clinical development, it remains to be determined how such engineered molecules will compare to naturally occurring antibodies in terms of pharmacokinetics, safety profiles, and the potential for inducing ADA responses in humans.

Currently the majority of bnAbs in clinical development are being evaluated as IgG1. Whether bnAbs expressed as IgA, IgM, or other IgG subclasses exhibit enhanced neutralization and/or effector activity is an area of active investigation. Indeed, several studies have demonstrated that certain bnAbs expressed as IgG3 have an increased ability to recruit ADCC and/or phagocytosis compared to their IgG1 counterparts (129, 130). These enhanced activities may be correlated with longer hinge length between the Fab and Fc domains and potentially higher affinity for FcγR-IIIA. Additional strategies in Fc engineering are being explored to increase antibody affinity to FcγRs expressed on innate immune cells and augment antibody-dependent effector activities. Modifications such as the AAA (S298A/E333A/K334A) or GASDALIE (G236A/S239D/A330L/I332E) mutations have been shown to increase antibody affinity for Fcγ-RIIIA and augment ADCC activity (131). Modifications of Fc-glycosylation patterns have also been demonstrated to enhance ADCC and ADCP activities (132). Additional evaluation in the NHP/SHIV model will be important to determine whether bnAbs expressed as alternative isotypes, IgG subclasses, or with modified Fc domains exhibit enhanced anti-viral protection *in vivo*, especially for therapeutic strategies in which activation of innate effector functions may be critical for eliminating infected cells. Additional understanding of the ability of bnAbs to inhibit cell-to-cell transmission versus cell-free transmission is also needed (133). While *in vitro* analyses have suggested that bnAbs may be less effective at inhibiting cell-to-cell transmission (134, 135), this may be dependent on the specific bnAb and/or epitope target, the virus strain and the degree of steric hindrance encountered at the virological synapse. Further insight into the mechanisms of cell-to-cell spread *in vivo* and the inhibitory potential of specific bnAbs will further inform optimal combinations for prevention and treatment strategies.

## Conclusions

HIV-1 bnAbs have shown significant promise for their potential use in the prevention and treatment of HIV-1 infection. Multiple bnAbs and bnAb combinations have been tested in human clinical trials and demonstrated favorable safety and pharmacokinetic profiles. The results from the first AMP efficacy trials have provided proof-of-concept that bnAbs can prevent acquisition of HIV-1, yet they also highlight the obstacles that must be addressed before bnAb passive immunization strategies can become integrated as a tool for standard clinical care. Combinations of highly potent bnAbs or engineered variants targeting multiple epitopes will likely be required to reliably inhibit the global diversity of circulating viruses encountered in nature and impede the development of resistance. Improving pharmacokinetic profiles and delivery methods such that bnAbs may be self-administered every 4-6 months by subcutaneous injection to attain persistent protective levels of serum neutralizing activity is a highly desired goal that the field continues to work towards. Additional efforts to optimize the Fc domain of bnAbs to enhance the activation of innate effector functions may further improve efficacy profiles, especially in the setting of therapeutic treatment of HIV-1 infection.

## Author Contributions

All authors listed have made a substantial, direct, and intellectual contribution to the work, and approved it for publication.

## Funding

This work was supported by the Bill and Melinda Gates Foundation Collaboration for AIDS Vaccine Discovery (CAVD) grant #OPP1146996 (MS) and the National Institute of Allergy and Infectious Diseases (NIAID) grant # UM1 AI069412 (SW).

## Conflict of Interest

The authors declare that the research was conducted in the absence of any commercial or financial relationships that could be construed as a potential conflict of interest.
